# Allergen immunotherapy using recombinant *Culicoides* allergens improves clinical signs of equine insect bite hypersensitivity

**DOI:** 10.3389/falgy.2024.1467245

**Published:** 2024-09-30

**Authors:** Anneli Graner, Ralf S. Mueller, Johanna Geisler, Delia Bogenstätter, Samuel J. White, Sigridur Jonsdottir, Eliane Marti

**Affiliations:** ^1^ Centre for Clinical Veterinary Medicine, Ludwig Maximilian University Munich, Munich, Germany; ^2^ Clinical Immunology Group, Division of Neurological Sciences, Department of Clinical Research—VPH, Vetsuisse Faculty, University of Bern, Bern, Switzerland; ^3^ Graduate School for Cellular and Biomedical Sciences, University of Bern, Bern, Switzerland; ^4^ Centre for Applied Innovation, York St John University, York, United Kingdom; ^5^ Institute for Experimental Pathology, Biomedical Center, University of Iceland, Keldur, Reykjavik, Iceland

**Keywords:** allergen immunotherapy, horse, insect bite hypersensitivity, *Culicoides*, recombinant allergens

## Abstract

**Introduction:**

Insect bite hypersensitivity (IBH) is an IgE-mediated allergic dermatitis of horses caused by bites of *Culicoides* spp., sharing some common features with human atopic dermatitis. Allergen immunotherapy (AIT) using *Culicoides* whole-body extracts has limited efficacy. This study aimed to evaluate AIT with a pool of major *Culicoides* recombinant allergens in a prospective, double-blinded, placebo-controlled study.

**Methods:**

The IBH lesion score was assessed during a pre-treatment year and first treatment year (May–October) in 17 horses and in May and July of a second treatment year. Nine horses were immunized subcutaneously 3× with a combination of nine r-allergens (20 μg each/injection) in alum and monophosphoryl lipid A (MPLA). Eight horses received a placebo. The immunization was repeated twice the following year. The specific antibody response to one of the AIT *Culicoides* r-allergens was assessed.

**Results:**

In the first treatment year, the decrease in average IBH lesion score was significantly larger in the AIT compared to the placebo group, with 67% of the AIT group and 25% of the placebo horses reaching >50% improvement of the average IBH lesion score. The response to the AIT was enhanced in the 2nd treatment year when 89% of the AIT vs. 14% of the placebo horses showed an improvement (*p* ≤ 0.01). IgG antibodies of all subclasses were induced, with IgG4/7 showing the most significant differences between groups. The post-AIT sera showed IgE blocking activity.

**Discussion:**

AIT using only a few injections of small amounts of r-allergens in alum and MPLA as immunomodulators seems a promising approach for the treatment of insect bite allergy.

## 1 Introduction

Equine insect bite hypersensitivity (IBH), also named *Culicoides* hypersensitivity (CH), is the most common skin disease in horses (1, 2), with an overall prevalence of 10%, and as high as 50% in horses imported from Iceland to continental Europe and some horse families (3–5). This highly pruritic allergic dermatitis is caused by an IgE-mediated hypersensitivity reaction to salivary proteins from *Culicoides* midges which secrete into the skin a cocktail of various proteins that facilitate their blood meal (6). Although the allergens differ, IBH has some common features with human atopic dermatitis (7) and is thus a natural model of allergy. There is presently no satisfactory treatment available for IBH.

Allergen immunotherapy (AIT) is the only disease-modifying treatment for IgE-mediated allergy (8). AIT influences the immune response at multiple levels to induce tolerance. It leads to an early reduction in the degranulation of mast cells, immune deviation from a Th2- to a Th1-response, induction of regulatory T cells (Treg) and B cells, and induction of allergen-specific IgG. These IgG antibodies inhibit mast cell activation through their binding to Fc*ε*RIIb and neutralize free allergens (8). An efficacious AIT is thus associated with a dampening of various proinflammatory responses while inducing strong regulatory counterparts. Similar mechanisms have been described in animals (9).

The effect of AIT as a treatment for IBH is controversial: while some studies showed a beneficial effect (10), placebo-controlled studies using commercial *Culicoides* extracts could not demonstrate significant differences between AIT and placebo (11, 12). A potential reason for this lack of response is the use of crude *Culicoides* whole-body extracts (WBEs) for AIT. Studies in human patients have shown a poor response when using crude, complex extracts in comparison to pure allergen preparations (13). Therefore, the specific *Culicoides* allergens associated with IBH were identified and 30 *Culicoides* proteins derived from salivary glands from *Culicoides sonorensis*, *Culicoides nubeculosus*, and *Culicoides obsoletus* have been produced as recombinant proteins (14). Using microarray technology, nine of these allergens were identified as major allergens for IBH in horses of various breeds including Icelandic horses (6).

To develop an immunization protocol for a preventive AIT, studies in non-allergic horses in Iceland compared various protocols using *Culicoides* r-allergens (15). The effects of the TLR-4 ligand monophosphoryl lipid A (MPLA), which is used in some AIT preparations for the treatment of allergic human patients (16) were also investigated in horses. *In vitro* and *in vivo* studies have shown that MPLA favors IL-10 while decreasing IL-4 production (15, 17) and that the immune response following subcutaneous or intralymphatic vaccination with r-allergens combined with alum and MPLA is similar (18).

The aim of this study was to investigate whether subcutaneous AIT of IBH-affected horses with a pool of major *Culicoides* r-allergens using alum and MPLA as adjuvants could improve clinical signs of IBH and gain an insight into the associated antibody response.

## 2 Material and methods

### 2.1 Study design

This was a prospective, double-blinded, placebo-controlled study. The protocol was approved by the Bavarian government under the number ROB-55-2-2532.Vet_02_20_20.

### 2.2 Study objects

In total, 17 privately owned Icelandic horses, all living in the same region near Munich in Bavaria, Germany, with ages ranging from 4 to 28 years were included in the study. All of them were diagnosed with IBH based on clinical presentation. All showed at least 2 years of typical skin lesions, strong pruritus, hair loss, and, in some cases, secondary bacterial infections, especially at the mane, tail, and dorsal and ventral midline during the insect season. All were protected from insect bites by blankets and/or stabling and/or the use of a topical lotion containing 25% benzyl benzoate. Thirteen horses were treated orally with an herbal solution with a repellent effect according to the manufacturer’s instructions (Ökozon, Einhorn, Osterholz-Scharmbeck, Germany). No changes in the treatment of the individual horses were made during the whole duration of the study.

### 2.3 Clinical evaluation

All horses were scored monthly by the same clinician and the owner from April until October during two subsequent years, consisting of the pre-treatment and the first treatment years (Supplementary Figure 1).

A standardized scoring system adapted from Olsen et al. (19) was used as an *IBH lesion score*. Briefly, each half of the horse's body was divided into 13 different zones, such as the head, hind legs, and neck. (19). In each zone, the clinical lesions such as crusts, lichenification, or excoriations were scored on a scale from 0 = healthy skin with no lesions to 3 = severe clinical lesions. The total score was calculated by adding the individual scores.

To determine a *pruritus score*, each horse owner had to score her/his horse twice weekly every fourth week during the insect season at the same time of the day using a modified scoring system by Friberg and Logas (20). Briefly, the owner had to observe the horse for 15 seconds (s) and record every pruritic act such as rolling, biting, stamping, scratching, or head shaking. Every act over a period of 15 s was awarded one point. The same occurred during the second subsequent period of 15 s in the same week. The sum of the points from both observations formed the monthly pruritus score.

In addition, the owner assessed the *overall hair coat and skin quality* monthly. The score was either 0 (healthy and normal), 1 (mild changes), 2 (mild to moderate changes), 3 (moderate changes), or 4 (severe changes).

### 2.4 Blood sampling

In the first treatment year, blood samples were taken by the clinician during each visit. Blood was taken from the left jugular vein into serum tubes (Vacuette, Greiner Bio-One GmbH, Frickenhausen, Germany), centrifuged (5000 rpm) for 10 min at room temperature, and the serum was kept frozen until further evaluation.

### 2.5 Treatment intervention

The horses were assigned to a placebo (*n* = 8) and a verum group (*n* = 9) to have balanced groups with regards to stables, the origin of the horse [born in Iceland and exported to Germany (IS) or born and living in Germany (DE)], age, and genders (Table 1). The horse owners and the clinician who immunized the horses and evaluated the clinical signs were all blinded.

**Table 1:** Horses used in the study.

Horse group	Mean age in years (range)	Male/female	Country IS/DE
Placebo	12.4 (4–23)	3/5	6/2
AIT	14.8 (5–28)	3/6	7/2

All horses belonged to the Icelandic breed. Some were born in Iceland and imported to German (IS) and the others were born and live in Germany (DE).

Horses were vaccinated subcutaneously into the left neck at the start of spring (March) and 4 and 16 weeks later. Nine horses were vaccinated with a pool of nine recombinant *Culicoides* allergens. The vaccine consisted of 20 μg each of eight *Culicoides obsoletus* (Cul o) recombinant (r-) allergens (Cul o 1P, Cul o 2P, Cul o 3, 5, 7, 8, 9, 11) and one *Culicoides nubeculosus* (Cul n) r-allergen (Cul n 4). All were produced in *Escherichia coli*, purified as described (6), and sterile filtrated before use. A mixture of 50 μg MPLA (MPLA-SM VacciGrade, InvivoGen, Toulouse, France, cat#vac-mpla2) and 500 μg aluminum-hydroxide-gel (Alhyhydrogel® 2%, InvivoGen, cat#vac-alu-50) was used as adjuvants as described previously (18). Allergens were chosen based on their relevance for IBH in Icelandic horses (6). The eight horses of the placebo group were vaccinated with 0.4 ml of sterile saline solution. All horses were examined prior to vaccination to ensure that the horses were healthy otherwise.

Based on the results from the first treatment year, the study was adapted and extended until July of the following year (second treatment year) on a slightly reduced number of horses with limited monitoring, only consisting of the assessment of the IBH lesion score in May and July by the clinician who was still blinded. In this second treatment year, the placebo (*n* = 7) and treatment horses (*n* = 9) were vaccinated in May and July as described above (Supplementary Figure 1).

### 2.6 Sensitization pattern to recombinant *Culicoides* allergens using a microarray

Allergen-specific IgE was determined in the sera taken during the summer of the pre-treatment year using the same microarray as described in Novotny et al. (6). The results are displayed as a percentage of IgE-positive horses for each single allergen.

### 2.7 Determination of allergen-specific serum IgE and IgG levels by ELISA

Serum levels of allergen-specific antibodies were measured in the sera taken at 10 different time points (March, April, May, June, July, August, September, and October in 2021, and May and July in 2022) in all horses using the most relevant *Culicoides* r-allergen included in the AIT (Cul o 8) and, for comparison, a *Culicoides* r-allergen not included in the AIT (Cul o 10). The enzyme linked immunosorbent assay (ELISA) was carried out as described previously (18, 21) but using 384-well plates (Immunolon 4HBX, Thermo Fisher Scientific, Rochester, NY, USA). Accordingly, volumes of 50 μl per well were used, except for blocking, where 80 μl were applied. For IgE detection, samples were run in duplicates, while triplicates were used for IgG determination. Briefly, plates were coated with the recombinant *Culicoides* r-allergens Cul o 8 and Cul o 10, expressed in insect cells and purified as described (18), diluted to 1 μg/ml in 0.2M carbonate–bicarbonate buffer, pH 9.4 (Thermo Fisher Scientific). Non-specific binding sites were blocked with 5% dried milk powder and 5% Tween 20 in phosphate buffered saline (PBS). Sera from the study horses as well as positive and negative control sera were diluted in blocking buffer, 1:10 for IgE and 1:800 for IgG determination, added to the plates, and incubated overnight at 4°C. After washing, anti-equine IgE [clone 3H10; (22)] or anti-equine IgG1, IgG4/7, or IgG5 were added (23). After incubation and washing, an alkaline phosphatase (AP) labeled goat anti-mouse IgG (Jackson ImmunoResearch, Ely, UK, Cat# 115-055-071, RRID: AB_2338535) was added. For pan IgG detection, an AP-labeled goat anti-horse IgG-Fc (Jackson ImmunoResearch Cat# 108-055-008, RRID: AB_2337493) was used. Plates were developed with p-nitrophenyl phosphate (PNPP) in 10% diethanolamine buffer, pH 9.8. Absorbance was measured at 405 nm. Blank values were subtracted and mean values were calculated.

### 2.8 Competitive inhibition ELISA

The ability of the horse sera to block IgE binding of IBH-horses to Cul o 8 was tested in an inhibition ELISA performed as previously described (21). Briefly, the ELISA plates were coated with Bac Cul o 8 and blocked as described above. Three pools of sera from the AIT or placebo group containing the same amount of serum from each of the horses within each group, taken before vaccination (March), after the third vaccination (August of the first treatment year), and in the second treatment year (July) were serially twofold diluted (1:10–1:160) and added to the plate. After incubation for 1 h at 37°C, serum from an IBH-affected horse with high IgE to Cul o 8 was added. The ELISA was then carried out as described above. The percentage of inhibition for each dilution of the pre- and post-vaccination serum pools was calculated.

### 2.9 Statistics

Graphs and statistical analyses were done in GraphPad Prism 10.2.2 (www.graphpad.com). As the data were not always distributed normally (Shapiro–Wilk Test), means and standard deviation or medians and interquartile range were plotted. Clinical lesion score, pruritus, and owner assessment scores were calculated for each month.

**Figure 1:**
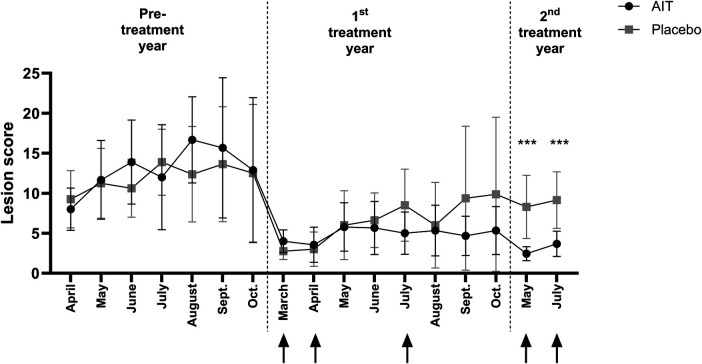
Monthly IBH lesion scores of the AIT and placebo groups shown as mean and standard deviation. The black dots symbolize the mean value of the horses in the AIT group (*n* = 9) and the gray boxes the mean value of the placebo group (*n* = 8). Vaccinations are indicated by black arrows. ***p < 0.0001 in the *t*-test comparing the groups at the single time points.

**Figure 2:**
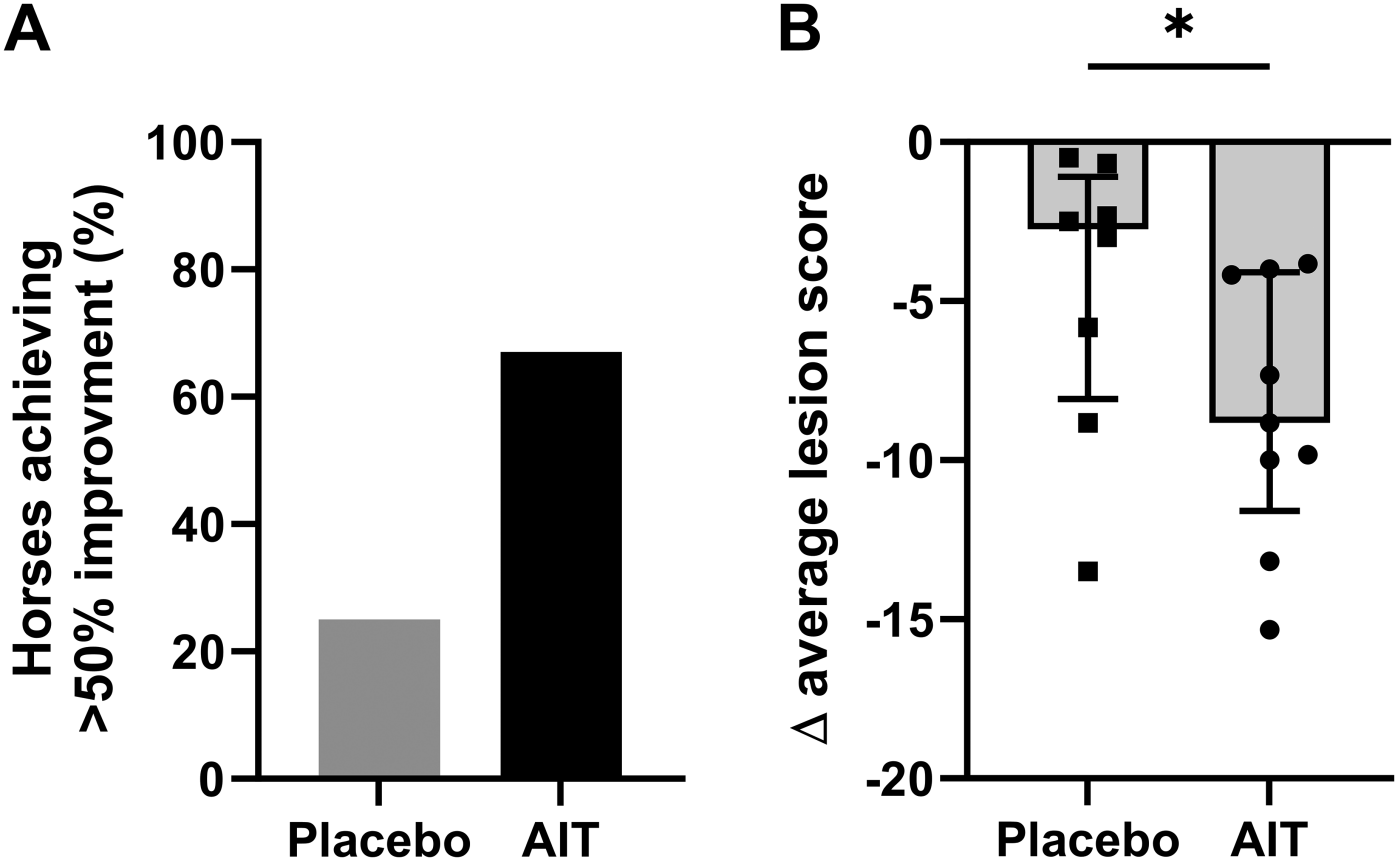
Response to AIT in the first treatment year. **(A)** Percentage of horses reaching >50% improvement in the average lesion score (May–October) compared to the pre-treatment year. **(B)** Comparison of the difference (Δ) in average lesion score of the pre-treatment and first treatment year between the placebo and AIT groups. Delta average lesion scores shown as median with interquartile range for each group. *p < 0.05 in the Mann–Whitney *U*-test.

**Figure 3:**
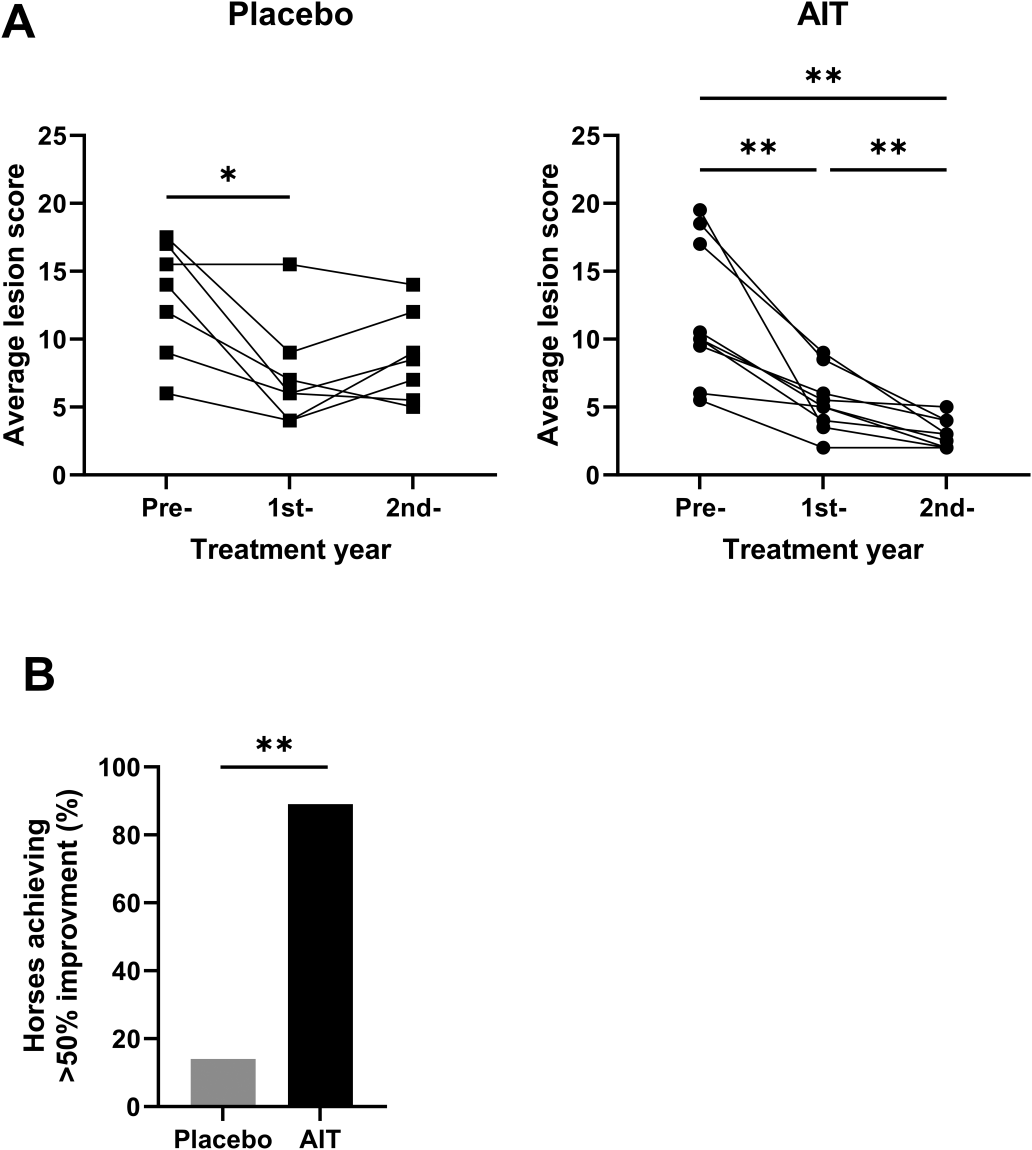
Response to AIT in the second treatment year. **(A)** Changes in the average lesion scores (May and July) between the pre-, first, and second treatment years in the placebo and AIT groups. A paired ANOVA with the Holm–Sidak multiple comparison test was used to compare the three time points. *0.01 ≥ *p* ≤ 0.05; **p < 0.01. **(B)** Percentage of horses reaching >50% improvement in the average lesion score (May and July) in the second treatment year compared to the pre-treatment year. **p < 0.01 in the two-sided Fisher’s exact test.

A comparison of the scores between the AIT and placebo groups was carried out for each month using a *t*-test (Figure 1). The response to treatment was assessed in analogy to Fettelschoss-Gabriel et al. (24). The average lesion score during the IBH season (May–October) was calculated for each horse. The percentage of horses achieving >50% improvement of the mean clinical lesion score was determined and compared between the AIT and placebo groups using a two-sided Fisher's exact test (Figure 2A). The same procedure was used to assess the treatment response in the second treatment year by using the average lesion scores of May and July of the pre- treatment and second treatment years (Figure 3B). Furthermore, for each month, the difference in scores between the treatment and pre-treatment years (Δ lesion score) was calculated and subsequently, the average of the Δ lesion scores (May–October) from the horses in the AIT group was compared to those treated with placebo using the Mann–Whitney test (Figure 2B). Average IBH lesion scores of May and July of the pre-, first, and second treatment years were calculated for each horse and compared using a paired ANOVA test with the Holm–Sidak correction for multiple comparisons (Figure 3A). Owner general assessments and pruritus were compared similarly. Furthermore, Spearman’s rank correlation coefficient between the IBH lesion score and the owners’ skin assessment and pruritus scores was calculated.

Statistical analysis of the serum antibody levels was performed using an RStudio software package (version 2023.06.1 + 524; www.r-studio.com). To test the global effect of the factors “group” (AIT/placebo) and “time point” on the antibody levels, a repeated measures analysis of variance was performed. Given the non-normally distributed data, a non-parametric test method according to Brunner et al. was used (25). The NparLD software package was used to analyze the individual antibody class levels to the individual *Culicoides* recombinant allergens Cul o 8 and Cul o 10. The NparLD software package employs robust rank-based methods for analyzing longitudinal data in factorial settings (26). ANOVA-type test statistics with Box approximation (ANOVA test) were calculated to assess group and time effects, as well as their interactions.

*p*-values ≤0.05 were considered significant.

## 3 Results

The monthly IBH lesion scores of the AIT and placebo groups over the complete duration of the study are shown in Figure 1.

### 3.1 IBH lesion score in the first treatment year

In both the placebo and treatment group, there was a reduction in the IBH lesion score in the treatment year compared to the pre-treatment year. There were no significant differences between the groups for any of the single time points in the pre- and first treatment years, although, in September and October, the mean IBH score started to diverge between the groups (Figure 1). Assessment of the IBH lesion score showed that 67% of the AIT and 25% of the placebo horses reached >50% improvement in the first treatment year (Figure 2A, ns). The decrease (Δ) in the average IBH lesion score between the treatment and pre-treatment year was significantly larger in the AIT compared to the placebo group (median = −8.83 in the AIT vs. −2.75 in the placebo group, *p* < 0.05; Figure 2B).

### 3.2 IBH lesion score in the second treatment year

The AIT effect was more pronounced in the second treatment year. Both in May and July, the IBH lesion score was significantly lower in the AIT compared to the placebo group (Figure 1). In the placebo group, the average IBH lesion score slightly increased from the first to the second treatment year (Figure 3A, ns). In contrast, the average IBH lesion score in the AIT group significantly decreased between the first and second treatment years (Figure 3A) and the variability of the scores was lower in the AIT group compared to the placebo group. Accordingly, 89% of the AIT horses but only 14% of the placebo horses achieved >50% improvement in the second compared to the pre-treatment year (Figure 3B, *p* < 0.01).

### 3.3 Owner assessment

**Figure 4:**
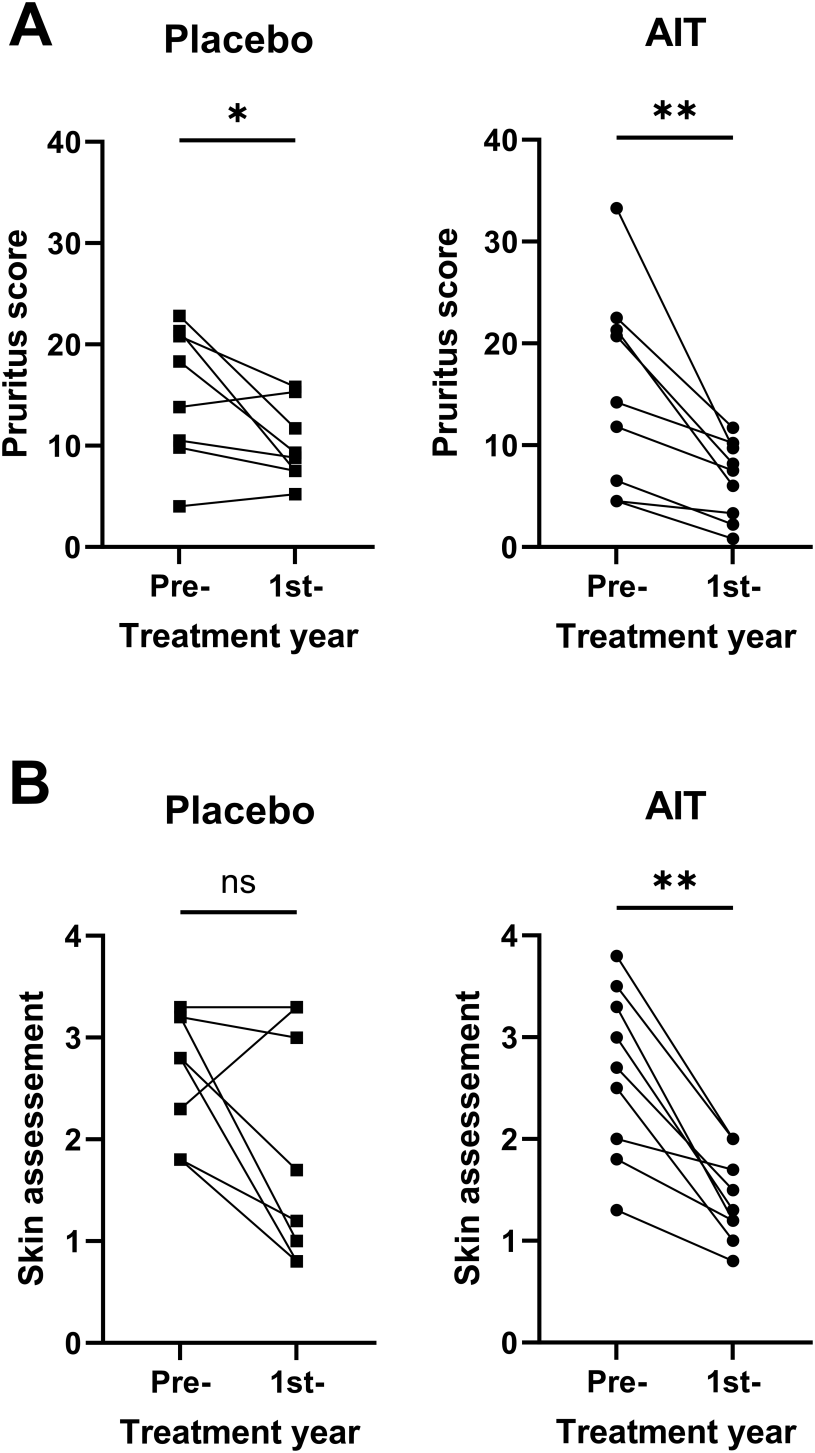
Owner assessment of placebo and AIT-treated horses in the pre-treatment and first treatment years. **(A)** Comparison of the mean pruritus scores (May–October) and **(B)** of the skin assessment, using the Wilcoxon paired test. *0.01 ≥ *p* ≤ 0.05; **p < 0.01.

The *pruritus score* decreased significantly in the treatment compared to the pre-treatment year in both the placebo and AIT groups, but the difference was more pronounced in the AIT group (Figure 4A). Owner assessment of the *hair coat and skin* showed a significant reduction of the score in the AIT group in the treatment compared to the pre-treatment year (*p* < 0.01). A reduction in the score was observed in all AIT horses (Figure 4B). In the placebo group, there was much more variability in score changes between the pre-treatment and treatment years. The difference between the treatment and pre-treatment years was not significant in the placebo group. Direct comparison between the AIT and placebo groups for pruritus or haircoat and skin scores did not result in any significant differences between the groups.

There was a strong correlation between the owner skin assessment and the IBH lesion scores (Spearman’s rank correlation coefficient = 0.74, *p* < 0.0001), while the correlation between the pruritus and the IBH lesion scores was moderate (0.49, *p* < 0.0001).

### 3.4 Sensitization pattern to recombinant *Culicoides* allergens using a microarray

**Figure 5:**
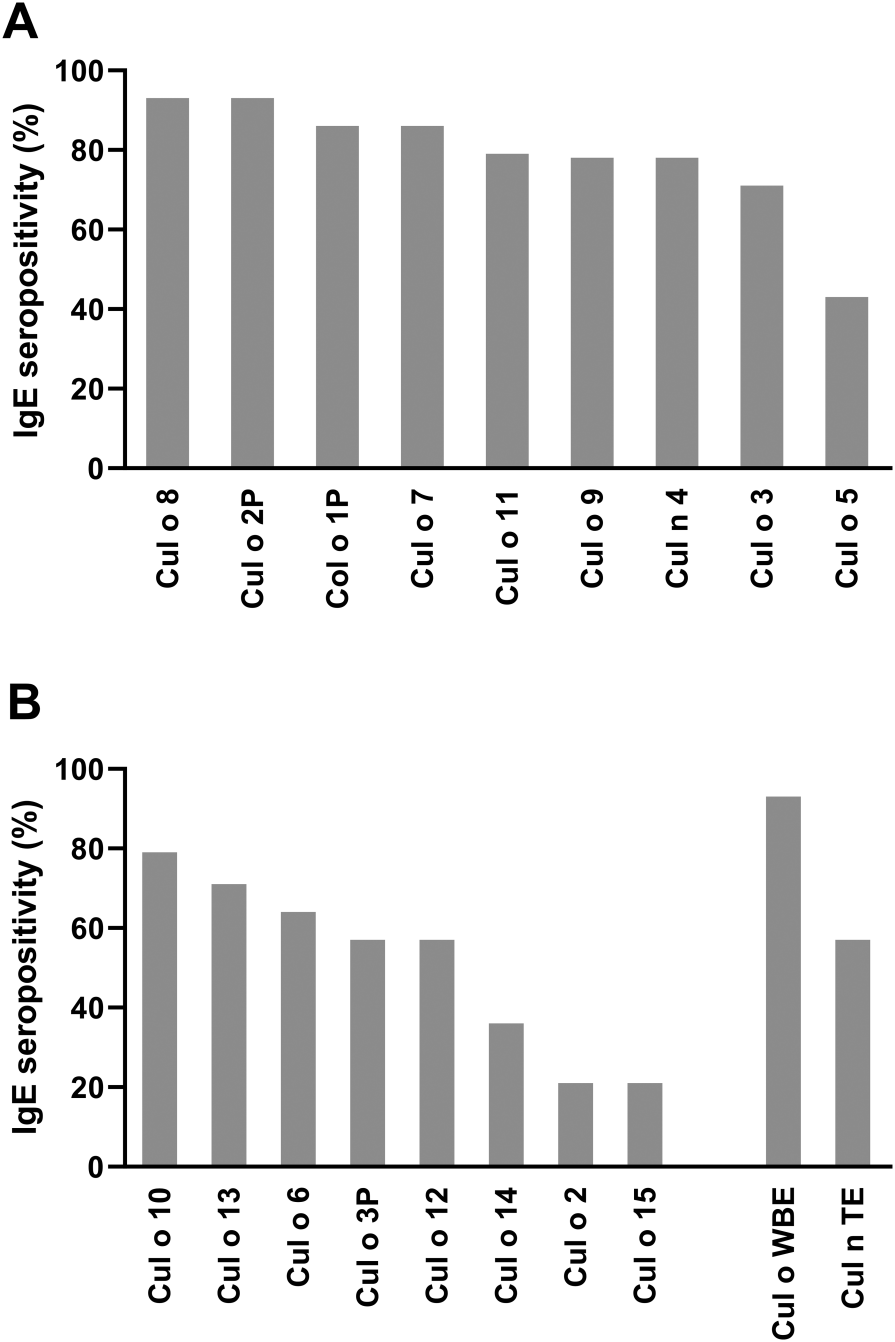
IgE seropositivity on the *Culicoides r-*allergens tested by microarray profiling. **(A)** *Culicoides* r-allergens included in the AIT. **(B)** *Culicoides obsoletus* r-allergens not included in the AIT as well as Cul o WBE and Cul n thorax extract (TE).

In the summer of the pre-treatment year, between 71% and 93% of the horses had positive IgE values to the single *Culicoides* allergens included in the vaccine, except for Cul o 5, where only 43% of the study horses had a positive IgE result (Figure 5A). The horses also showed varying sensitization patterns to the other eight *Culicoides obsoletus* allergens of the microarray not included in the vaccine (Figure 5B).

A similar pattern of sensitization to the vaccine allergens was observed in the AIT and placebo groups, except for Cul o 5 to which only 20% of the AIT vs. 80% of the placebo group were IgE positive (Supplementary Figure 2). The AIT horses were sensitized to a mean number of 6.9 of the nine vaccine allergens (range 0–9). The placebo horses were sensitized to almost the same number of allergens (mean = 7.4 allergens, range 2–9 allergens).

### 3.5 Allergen-specific serum IgE and IgG antibodies

**Figure 6:**
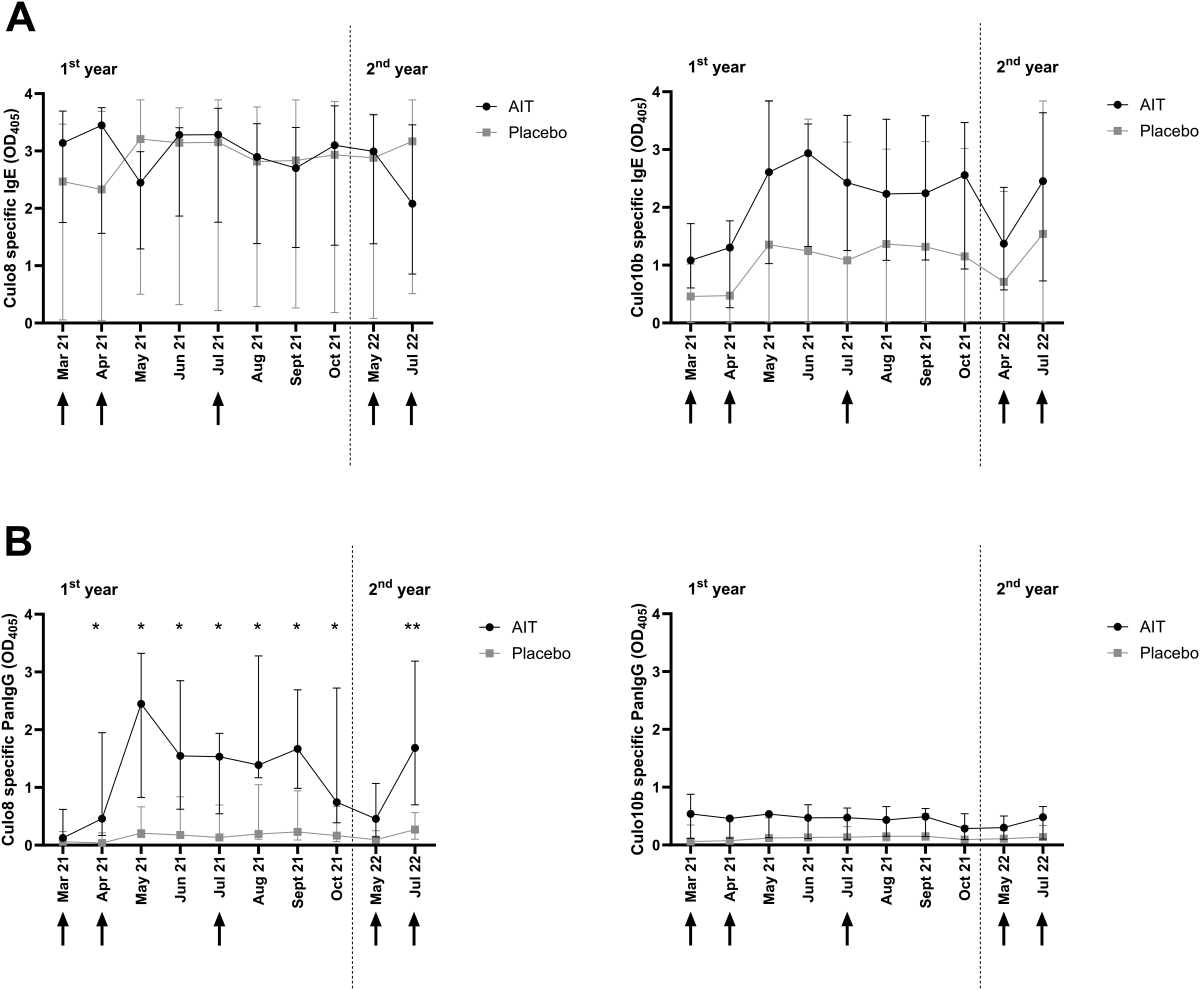
Antibody levels in the first and second treatment year, shown as median and interquartile range, specific for the AIT allergen Cul o 8 and Cul o 10, a *Culicoides* obsoletus allergen not included in the AIT. Vaccinations are indicated by black arrows. **(A)** IgE and **(B)** pan IgG levels in the sera of the AIT-treated (black circles) and placebo (gray squares) horses. Comparisons between the groups at the single time points were conducted with a Wilcoxon signed rank test. *0.01 ≥ *p* ≤ 0.05; **p ≤ 0.01.

As shown for the AIT allergen Cul o 8 (Figure 6A), IgE serum levels did not differ significantly between the placebo and AIT groups (Brunner–Langer model). However, there was a significant variation over time in Cul o 8-specific IgE levels (*p* ≤ 0.05) as well as a significant (*p* ≤ 0.05) interaction between group and time: changes over time in IgE levels showed a different pattern in the AIT group and in the placebo group. In the AIT group, IgE levels to Cul o 8 had already increased 4 weeks after the first immunization, i.e., in April, and decreased in May, after the second immunization. In the placebo group, IgE started to increase only in May, i.e., 1 month later than in the AIT group, probably because of exposure to *Culicoides*. Later, median IgE levels remained similar in both groups until May of the next year, but then they decreased in the AIT group and increased in the placebo group. IgE levels to Cul o 10, an allergen not included in the vaccine, were higher in the AIT group but the difference was not significant. Cul o 10-specific IgE also varied over time (*p* ≤ 0.01) but the changes were parallel in both groups (Figure 6A) and thus there was no significant interaction between group and time in the Brunner–Langer model.

IgG levels increased after immunization in the AIT group (Figure 6B), and, consequently, IgG levels to Cul o 8 were significantly higher in the AIT compared to the placebo group (Brunner–Langer model, *p* < 0.05). IgG to Cul o 8 varied significantly over time (*p* < 0.001) and the interaction between group and time was significant (*p* < 0.05). IgG levels to Cul o 10 did not differ significantly between the groups, as shown in Figure 6B. They only varied slightly over time (*p* < 0.01) and no interactions between group and time were observed.

**Figure 7:**
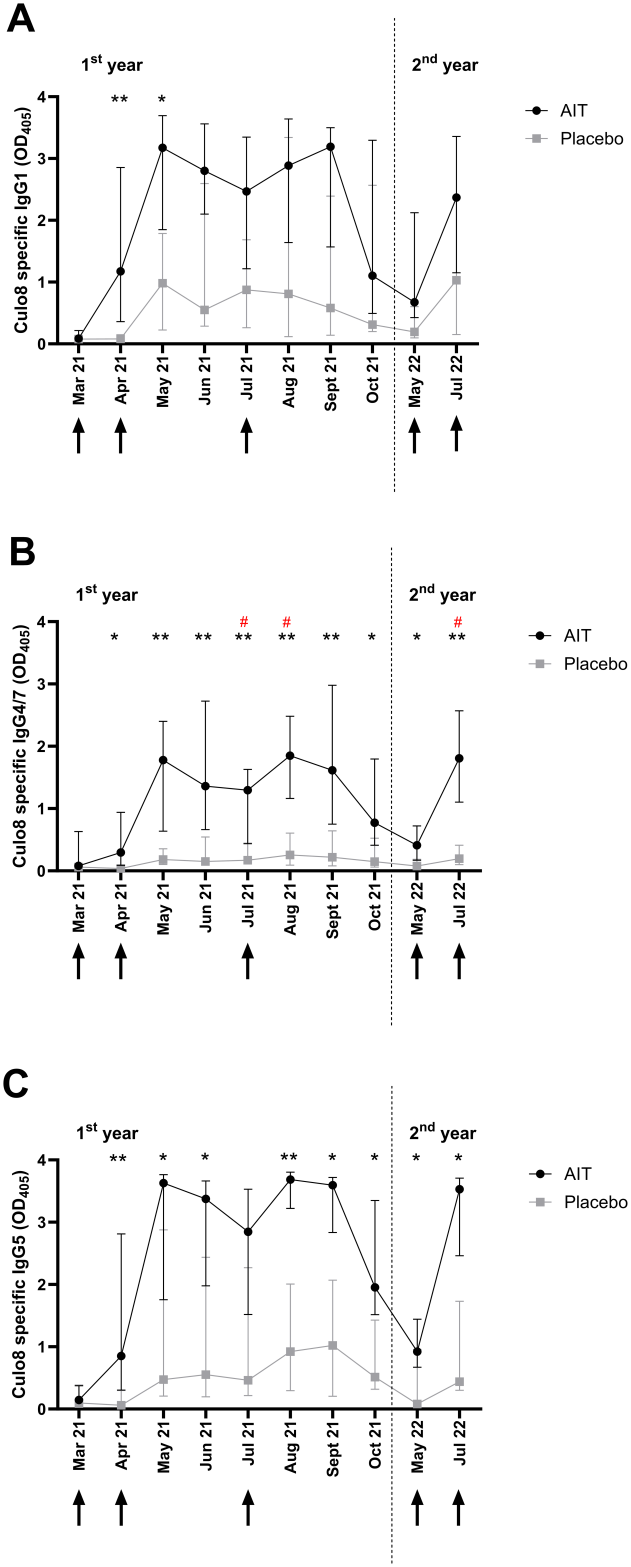
IgG subclasses to the AIT allergen Cul o 8 in the first and second treatment year. Median and interquartile ranges of IgG1 **(A)**, IgG4/7 **(B)**, and IgG5 **(C)** antibody levels in the sera of horses treated with AIT (black circles) and placebo (gray squares). Vaccinations are indicated by black arrows. Comparisons between the groups at the single time points were conducted with a Wilcoxon signed rank test. *0.01 ≥ *p* ≤ 0.05; **p ≤ 0.01. ^#^Significance (*p* ≤ 0.05) after Bonferroni correction for multiple comparisons.

The IgG subclass response was investigated for Cul o 8 (Figure 7). Significant effects of group (*p* < 0.05) and time (*p* < 0.00001) were observed for the three tested subclasses IgG1, IgG 4/7, and IgG5. After an increase following the immunizations, all subclasses decreased until the following May. The immunization in May 2022 resulted again in an increase in all IgG subclasses. Significant interactions between the treatment group and time were observed for IgG1 and IgG4/7 (*p* < 0.05). When comparing the antibody levels to Cul o 8 at the single time points between the AIT and placebo groups, IgG1 levels were only significantly higher in the AIT compared to the placebo group in April and May, while IgG4/7 levels were significantly higher in the AIT group at all times points following the first AIT. This was similar for IgG5, except for July in the 1st treatment year. Interestingly, IgG4/7 was the only antibody class where the difference between the groups reached significance after Bonferroni correction for multiple comparisons.

### 3.6 Inhibition activity of the horse sera following AIT

**Figure 8:**
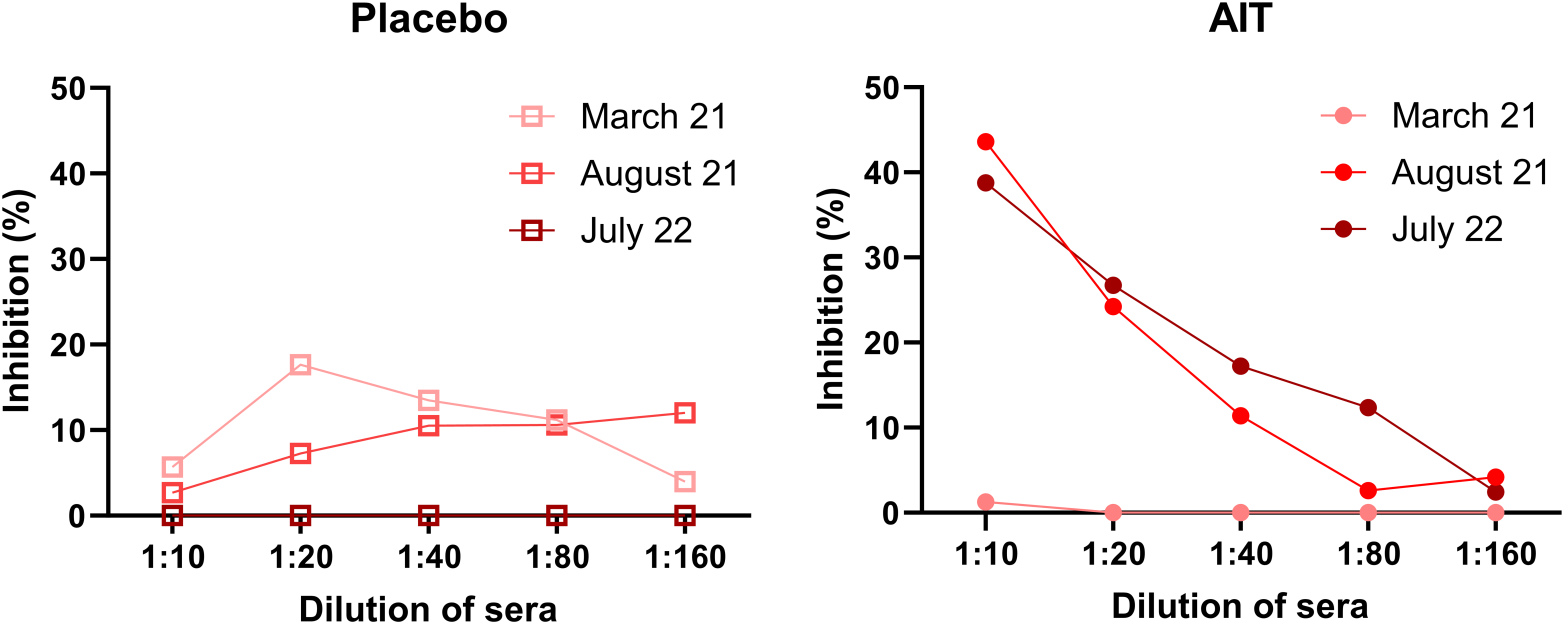
Inhibition of IgE binding to Cul o 8 with pools of sera from the AIT-treated and placebo horses. Mean percent inhibition by pre- and post-vaccination sera from the two groups, diluted 1:10–1:160 applied to an ELISA plate coated with Cul o 8, prior to adding serum from an IBH IgE-positive horse at a dilution of 1:5. Empty boxes represent pools from the placebo horses and full circles pools from the AIT-treated horses. March 21 = pre-immune sera; August 21 = sera taken 1 month after the third immunization in the first treatment year; July 22 = sera taken in the second treatment year.

The blocking activity of the sera was investigated for the allergen Cul o 8. AIT-induced antibodies were able to block IgE binding to this allergen. The blocking activity of the serum pool of the AIT group increased from 1.5% in the pre-immune sera to 43% after the third immunization and was similar in July of the second treatment year. The blocking activity of the serum pools taken after the AITs was dilution-dependent, i.e., it decreased almost linearly between the 1:10 and 1:160 serum dilutions. Some blocking activity was seen with the serum pools of the placebo group, but it was random and not dilution-dependent (Figure 8).

## 4 Discussion

The aim of this study was to investigate the effect of AIT on the treatment of IBH by using a pool of the most relevant *Culicoides* r-allergens (6), instead of a crude *Culicoides* whole-body extract which showed no beneficial effect in placebo-controlled studies (11, 12). We decided to use a pool of major *Culicoides* r-allergens instead of a patient-tailored *Culicoides* r-allergen AIT because of the higher complexity and costs of the latter, and consequently also a higher potential of a single pool of *Culicoides* r-allergens for future commercial development. The panel of *Culicoides* r-allergens consisted of eight of the nine major allergens identified by Novotny et al. (6), and of an additional *Culicoides nubeculosus* allergen, Cul n 4, which was also a major allergen for Icelandic horses (27). The major *Culicoides* allergen Cul o 10 could unfortunately not be included in the AIT because of technical problems during production. Serum IgE profiling of the study horses using the same microarray as described in Novotny et al. (6) confirmed the relevance of the r-allergens included in the AIT as, except for Cul o 5, >70% of the study horses had positive IgE values to the single *Culicoides* r-allergens included in the vaccine (Figure 5A). Testing on the microarray also confirmed the higher relevance of *Culicoides obsoletus* vs. *Culicoides nubeculosus* allergens, as >90% of the horses were IgE-positive to *Culicoides obsoletus* vs. only 57% to *Culicoides nubeculosus* extract, supporting the decision to preferably use Cul o allergens for the AIT. The horses also showed varying degrees of sensitization to the additional eight *Culicoides obsoletus* r-allergens of the microarray that were not included in the vaccine (Figure 5B). However, except for the leucine-rich domain (Cul o 12) and the DUF4803 superfamily (Cul o 10), all *Culicoides obsoletus* allergens that were major allergens in our study horses were represented by at least one r-allergen of the given allergen family in the AIT vaccine.

The AIT protocol used here was developed based on previous studies showing that intralymphatic immunotherapy (ILIT) with 3 injections had the same efficacy as 54 s.c. injections over 3 years (28), but that in horses, 3 s.c. injections of r-allergens induced a similar immune response as three injections into the lymph node (18). In the first treatment year, both the AIT and placebo horses improved in their IBH lesion score compared to the pre-treatment year but the AIT-treated horses had a significantly larger reduction in the average clinical lesion score than the placebo group, and this with only three subcutaneous injections. Consequently, more AIT-treated horses achieved a >50% improvement in the average clinical lesion score. The clinical improvement was also evident in the owners’ assessment of skin quality and pruritus, as a more consistent improvement of the pruritus and skin assessment scores was found in the AIT compared to the placebo group. However, significant differences between the groups were only found when using the IBH lesion score from Olsen et al. (19), possibly because this scoring was more precise than the owner assessment. Nonetheless, the owners’ skin assessment correlated well with the IBH lesion score.

The study was originally designed to last over 2 years, with a clinical IBH scoring of the patients during one pre-treatment and one treatment season. Because a first analysis of the data at the end of the original study showed that differences between the groups were starting to appear in September we tried to extend the study for a second treatment year. This was only possible until July of the following year and without the owner assessments. Both the clinician and horse owners had not been unblinded. Interestingly, in the second treatment year, the IBH lesion score further decreased in the AIT group, while it remained almost similar to the preceding year in the placebo group (Figure 3A). This resulted in significant differences between the groups in May and July (Figure 1). Almost 90% of the AIT-treated horses achieved a >50% improvement in their clinical lesion score in the second treatment year, while only 14% of the placebo horses showed such an improvement, illustrating the excellent response to the AIT in the second treatment year. Based on the results of this study, AIT should be carried out over at least 2 years, as indicated in other species. The AIT was well-tolerated with no side effects other than local swelling at the injection site in a few horses.

To gain insight into the antibody response induced by this AIT protocol, IgE and IgG subclasses were measured against two allergens, Cul o 8, one of the most relevant allergens included in the AIT, and Cul o 10, which is also a major allergen for IBH but could not be included in the AIT. As to be expected, the AIT resulted in an increase in Cul o 8-specific IgG that was not observed for Cul o 10. Horses have seven IgG subclasses, with different functions (29) but not all can be measured as a single subclass (23, 29). Based on our previous studies (30, 31), we measured allergen-specific IgG1, IgG4/7, and IgG5. All three subclasses were induced by the AIT but the most significant differences between the AIT and placebo groups were seen for IgG4/7. This can be explained by the fact that almost no IgG4/7 was induced by natural *Culicoides* exposure, while IgG1 and IgG5 also increased in the placebo group, confirming results of a previous study that showed that allergen-specific IgG1 and IgG5 are increased in IBH-affected horses (31). IgE levels specific for Cul o 8 and Cul o 10 did not differ significantly between the groups but the time course of IgE antibodies against Cul o 8 was different between the AIT and placebo groups, decreasing in the AIT group at the end of the study, while they increased in the placebo group. This late decrease of serum IgE after AIT is in accordance with the AIT mechanisms described in human patients (32). Furthermore, blocking antibodies (Figure 8) were induced by the AIT, confirming the beneficial effect in the treated patients. Ideally, natural Cul o 8 should have been used to assess the blocking activity of the sera. As natural Cul o 8 is not available, we used, as for the other ELISAs, recombinant insect cell-expressed Cul o 8, which should be the closest to the natural protein.

This study has some limitations. The data collected are partly based on personal perceptions and are dependent on factors that cannot be influenced, such as the weather, i.e., the activity of *Culicoides* at the time of the evaluation of the patients. For this reason, as done in this study, it is of utmost importance to include placebo horses living in the same environment. It is known that *Culicoides* prefer warm, humid weather with little wind and are particularly active at dusk (33, 34). The placebo effect observed in our study could thus potentially also be due to climatic differences between the pre-treatment and first treatment year, as the summer of the first treatment year was particularly rainy and cold, limiting the activity of the midges. Furthermore, a true placebo effect due to closer care of the horses is also possible. A placebo effect is often observed in AIT studies, as also observed in previous IBH studies (11, 12, 24). Another limitation of this study is that the major allergen Cul o 10 was not included in the AIT because of technical difficulties. This allergen was an important allergen for our horses, as IgE serology performed on the microarray showed that 80% were sensitized to Cul o 10 (Figure 5B). The relatively small number of horses included in the study did not allow for investigation of possible effects of the origin of the horse (i.e., born in Iceland or in Germany) or of the age of the horse on the response to treatment. Nevertheless, these factors should not have influenced the results of our study as the treatment groups were balanced with regards to the origin of the horse, age, and stable.

This is the first placebo-controlled study which shows a beneficial effect of AIT for treatment of IBH. It indicates that a few injections of a pool of *Culicoides* r-allergens in combination with Alum and MPLA as immunomodulators is a promising approach for the treatment of this allergy.

## Data Availability

The raw data supporting the conclusions of this article will be made available by the authors, without undue reservation.

## Supplementary material

Supplementary Figure 1 Outline of the study over the 3 years, figure made with Biorender.

Supplementary Figure 2 IgE seropositivity on the *Culicoides* r-allergens included in the vaccine, tested by microarray profiling in the AIT and placebo groups. Except for Cul o 5 (<0.05, Pearson’s chi-square test), there were no significant differences in the distribution of positive and negative test results between the AIT and placebo horses.
