# Supplementary figures and images for: Allergen immunotherapy using recombinant Culicoides allergens improves clinical signs of equine insect bite hypersensitivity
Source: Front Allergy. 2024 Sep 30;5:1467245. doi: 10.3389/falgy.2024.1467245 (PMC11471737; doi:10.3389/falgy.2024.1467245)

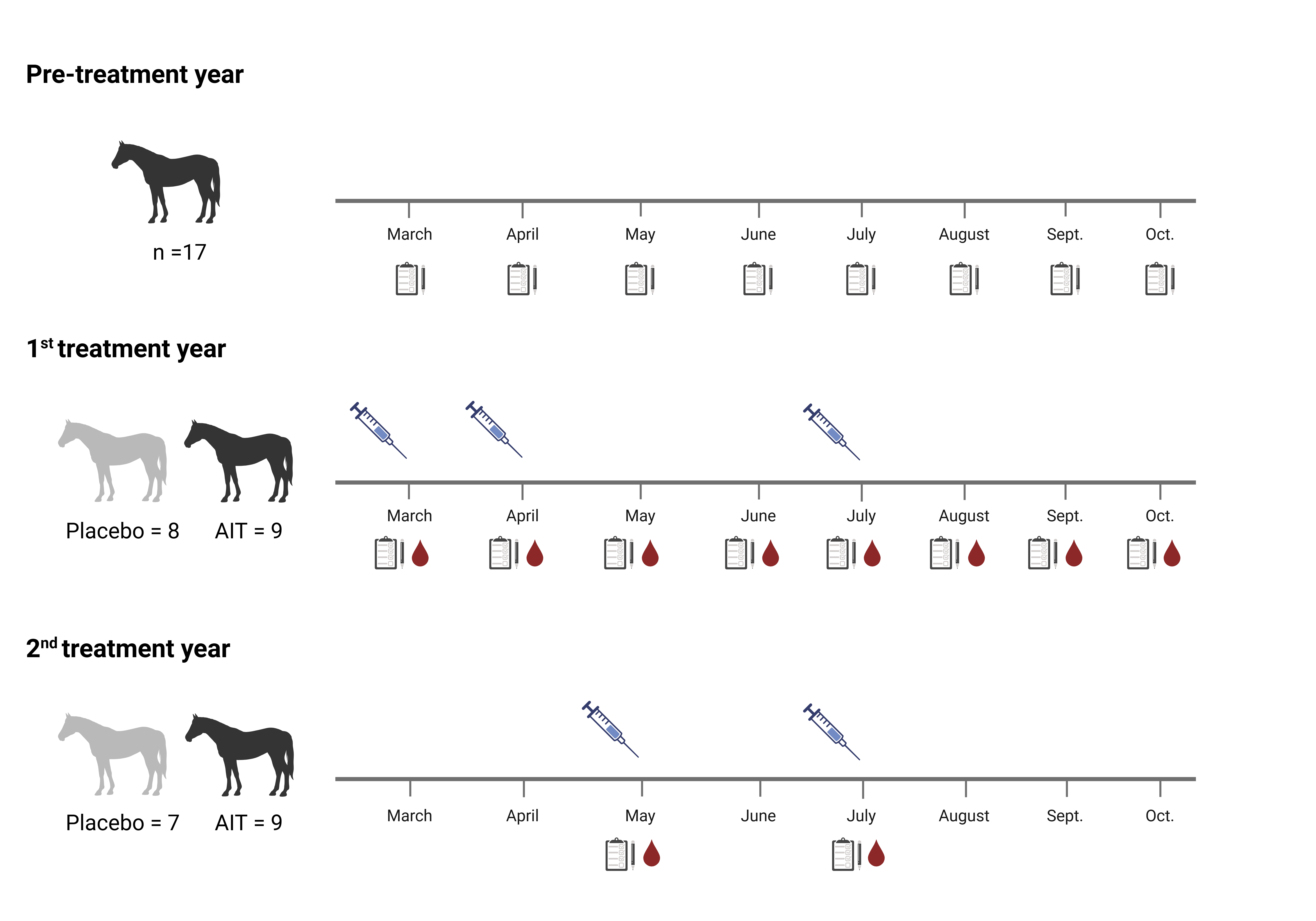

Supplement: Supplementary Figure 1 — Outline of the study over the 3 years, figure made with Biorender. [file Image1.jpeg]

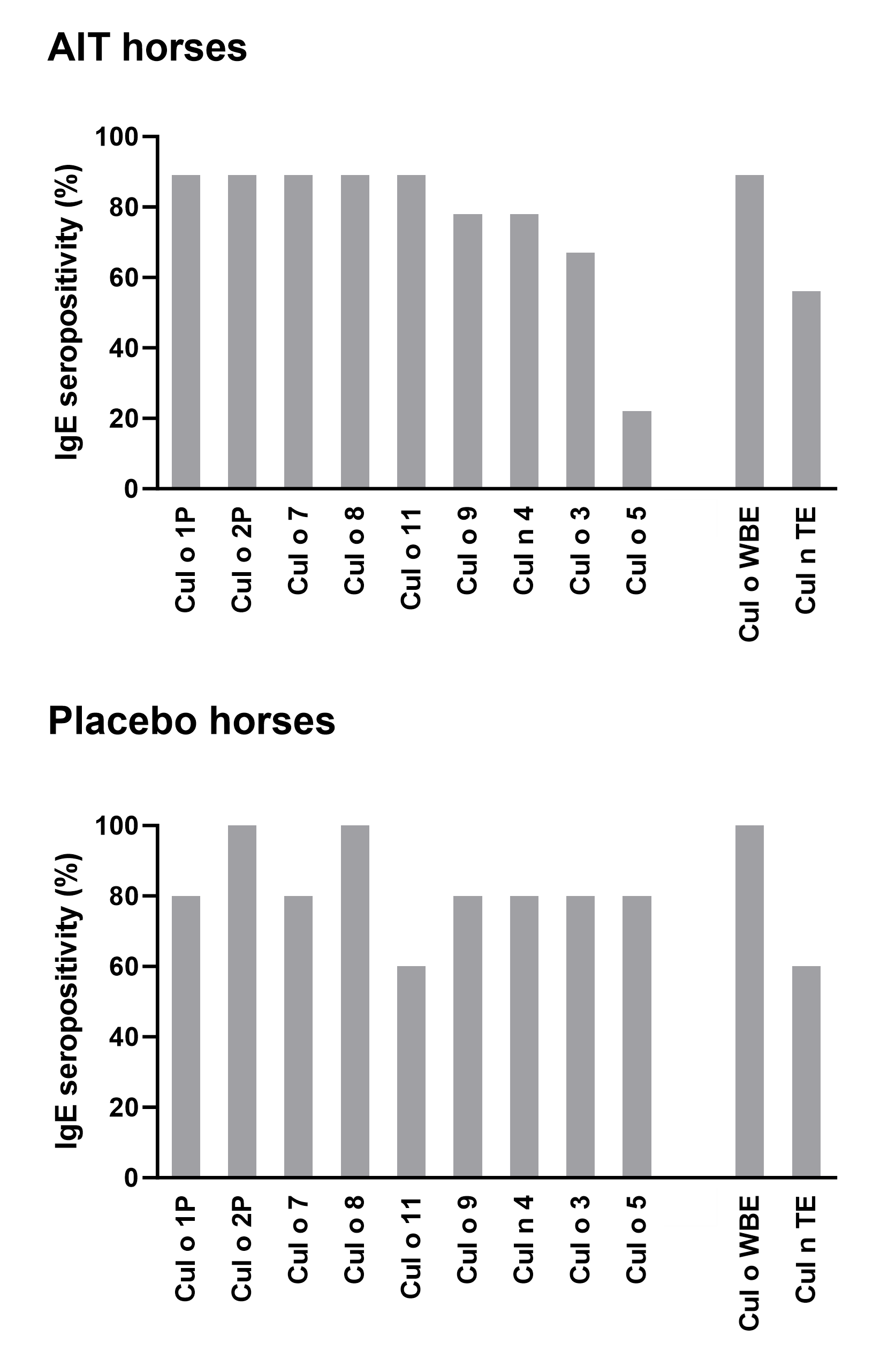

Supplement: Supplementary Figure 2 — IgE seropositivity on the Culicoides r-allergens included in the vaccine, tested by microarray profiling in the AIT and placebo groups. Except for Cul o 5 (<0.05, Pearson’s chi-square test), there were no significant differences in the distribution of positive and negative test results between the AIT and placebo horses. [file Image2.tif]
